# Time of Administration of Acute or Chronic Doses of Imipramine Affects its Antidepressant Action in Rats

**DOI:** 10.5334/jcr.156

**Published:** 2018-05-10

**Authors:** Hiroshi Kawai, Natsumi Kodaira, Chika Tanaka, Takuya Ishibashi, Naomi Kudo, Yoichi Kawashima, Atsushi Mitsumoto

**Affiliations:** 1 Faculty of Pharmacy and Pharmaceutical Sciences, Josai University, 1-1 Keyakidai, Sakado, Saitama 350-0295, JP; 2 Faculty of Pharmaceutical Sciences, Josai International Universit, 1 Gumyo, Togane, Chiba 283-8555, JP

**Keywords:** Chronopharmacology, Imipramine, Forced swim test, Noradrenaline, α-Adrenergic receptor

## Abstract

The pathogenesis and therapeutics of depression are linked to the operation of the circadian system. Here, we studied the chronopharmacological action of a tricyclic antidepressant, imipramine. Male adult Wistar–Hannover rats were administered imipramine acutely or chronically in the morning or in the evening. The antidepressant action of imipramine was analyzed using the forced swim test (FST). A single dose of imipramine (30 mg/kg) in the morning, but not in the evening, reduced immobility and increased climbing in the FST. The plasma concentrations of imipramine and its metabolite, desipramine, were slightly higher in the morning than in the evening, which might explain the dosing time-dependent action of imipramine. Next, we analyzed the effect of chronic imipramine treatment. Rats received imipramine in the morning or in the evening for 2 weeks. The morning treatment resulted in larger effects in the FST than the evening treatment, and was effective at a dose that was ineffective when administered acutely. The levels of brain α-adrenergic receptors tended to decrease after chronic imipramine treatment. Imipramine might interact with noradrenergic neurons, and this interaction might chronically alter receptor expression. This alteration seemed greater in the morning than in the evening, which might explain the dosing time-dependent action of imipramine.

## Introduction

The pharmacological action of many substances is dependent on the circadian system, and a drug’s dosing time can affect both its therapeutic and toxic effects [1,2,3,4,5]. Some drugs work better in the daytime, while other drugs work better at night. The best timing for a treatment depends on the chronopharmacological profile of the drug. Therefore, precise knowledge of the chronopharmacological profile of a drug is essential to provide effective medication.

Depression is strongly associated with the circadian system. Patients with depression often show a disturbance of the circadian rhythm of various physiological functions (e.g., a sleep disturbance). Depression and circadian disturbance may be physiologically linked and deteriorate synergistically [6,7,8]. However, the circadian system has been linked not only to the pathogenesis of depression but also to its therapeutics [9,10,11,12,13]. Wehr et al. reported that the circadian phase was advanced and desynchronized with the sleep-wake cycle in patients with depression, and that phase advance of the sleep-wake cycle exerted an antidepressant effect [10]. Phase entrainment by bright light in the morning is also effective to improve depressive symptoms [11,12]. Chronotherapy of depression by the combination of sleep deprivation, sleep phase advance, and bright light treatment induces rapid amelioration in patients with depression [13]. Some antidepressants can interact with and modify the biological clock [14,15,16,17,18,19]. In addition, some antidepressants show dosing time-dependent action in animal models [20,21] and in clinical settings [22,23]. However, the therapeutic significance of these actions remains to be clarified. Further research on the relationship between the circadian system and depression could lead to the development of an effective therapy for depression.

The forced swim test (FST) is one of the most common behavioral tests in rodents. Since the FST has high predictive validity, reliability, and robustness, it is useful for screening and analysis of antidepressant activity [24]. However, there is an inconsistency between the effects of antidepressants in the FST and those in clinical settings. A single dose or 2–3 injections of the antidepressant within 24 hours induce reduction of immobility indicative of antidepressant activity in the FST [25,26,27], whereas most antidepressants are ineffective when administered acutely or subacutely in clinical settings, usually taking several weeks to show therapeutic effects in patients [24]. Chronic antidepressant treatment alters brain function by regulating receptor expression levels, which might contribute to alleviating the depressive symptoms [28,29,30]. Those changes are not detectable in acute treatment. In order to analyze the biochemical alterations related to antidepressant activity, an animal model of chronic treatment would be necessary.

Several animal and clinical studies have reported the chronopharmacological action of antidepressants. Amitriptyline, fluvoxamine, and milnacipran show dosing time-dependent antidepressant activity in rodents [20,21]. However, these studies have used an acute treatment model with the FST. Several clinical studies analyzed the chronopharmacology of chronic antidepressant treatment [22,31,32]. However, no such analysis has been performed in animal models. In the present study, we aimed to develop a chronic animal model to analyze the chronopharmacological action of the antidepressant imipramine and to reveal the biochemical alterations that contribute to the chronopharmacological action of this antidepressant in this model. The present study analyzed the dosing time-dependent action of imipramine in an acute and chronic treatment model using the FST. We also analyzed the dosing time-dependent imipramine metabolism in the acute model and the dosing time-dependent effects of the chronic imipramine treatment on the brain levels of the α_1_ adrenergic receptor (α_1_AR) and α_2_ adrenergic receptor (α_2_AR). We hypothesized that the circadian fluctuation of imipramine metabolism may induce dosing time-dependent changes in the efficiency of the interaction between imipramine and the adrenergic system, a target system of imipramine. Repeated interaction caused by chronic imipramine treatment may induce a dosing time-dependent alteration of adrenergic receptors protein expression, which might contribute to the chronopharmacological antidepressant action of imipramine.

## Methods

### Chemicals

Imipramine and other chemicals were obtained from Wako Pure Chemical Industries (Osaka, Japan), unless otherwise described. Pentobarbital was obtained from Kyoritsu Seiyaku (Tokyo, Japan). Protease inhibitor cocktail and anti-β-actin antibody (A5441) were obtained from Sigma-Aldrich (St. Louis, MO, USA). Anti-α_2_-adrenergic receptor antibody (sc-28983) was obtained from Santa Cruz Biotechnology (Dallas, TX, USA). Anti-α_1_-adrenergic receptor antibody (ab137123), anti-rabbit immunoglobulin G (IgG) horseradish peroxidase-conjugated secondary antibody (ab97051), and anti-mouse IgG horseradish peroxidase-conjugated secondary antibody (ab79023) were obtained from Abcam (Cambridge, MA, USA).

### Animals

Male adult Wistar–Hannover rats (weighing 200–250 g) were obtained from CLEA Japan (Tokyo), and were maintained in an air-conditioned room at 24 ± 2°C with a 12/12-hour light/dark cycle (lights on at 7:00 a.m.). The rats had free access to food and water. The time of the day is expressed as the zeitgeber time (ZT), defined as follows: the light onset time (7:00 a.m. clock time) is ZT0; the dark onset time (7:00 p.m. clock time) is ZT12; and ZT24 is the ZT0 of the next day. Animal maintenance and treatments were in accordance with the general recommendations of animal protection legislation in Japan. All procedures were approved by the Institutional Animal Care and Use Committee of Josai International University (approval numbers: 32 and 57).

### Schedule for the analysis of the effects of acute imipramine treatment

One day before measurement, rats were exposed to the FST apparatus as described below. Twenty-three hours after the first exposure, rats were intraperitoneally administered 10, 20, 30, or 50 mg/kg of imipramine (as a hydrochloride salt) at 8:00 a.m. (ZT1) or 8:00 p.m. (ZT13). Control rats were administered saline. One hour after administration, the FST was carried out.

### Schedule for the analysis of the effects of chronic imipramine treatment

Rats were exposed to the FST apparatus as described below, and the drug treatment was started the next day. The rats were intraperitoneally administered saline or imipramine for 2 weeks. To equalize the number and the timing of the intraperitoneal injections among the groups, all rats were injected twice a day as shown in Table 1. Control rats were administered saline at ZT1 and ZT13; the morning group was administered imipramine at ZT1 and saline at ZT13; and the evening group was administered saline at ZT1 and imipramine at ZT13. Two days after the last administration, the rats were administered imipramine at a low dose (10 mg/kg) during the early light period (ZT1–3), and 1 hour after the administration, the FST was carried out. The rats were returned to their home cage after the FST, and they were sacrificed for biochemical analysis on the next day.

**Table 1:** Daily administration schedule for the analysis of chronic imipramine treatment.

Group	Administration time	
	ZT1	ZT13

Control	Saline	Saline
Morning (imipramine at ZT1)	Imipramine	Saline
Evening (imipramine at ZT13)	Saline	Imipramine

### Forced swim test (FST)

We used a modified FST according to the method of Detke et al. [26]. On the day before the first treatment of imipramine or saline, each rat was individually placed in a tank made of an opaque plastic cylinder (24 cm in diameter, 50 cm in height) filled with room-temperature water (24 ± 2°C) to a depth of 30 cm. The rat was left in the tank for 10 minutes, then removed from the cylinder, allowed to dry in a clean cage, and returned to its home cage. On the day of measurement, the rat was again placed in the tank of room-temperature water, and its behaviors were analyzed for 5 minutes: at regular 5-second intervals, trained observers recorded which of the following three behaviors was predominant: immobility, swimming, or climbing. The total counts for each behavior over the 5-minute test session were analyzed as the FST score. All tests were observed and scored by two or more trained observers to prevent scoring errors.

### Analysis of plasma imipramine concentration

Rats were administered intraperitoneally with 30 mg/kg of imipramine at ZT1 or ZT13. One hour after administration, the rats were anesthetized with pentobarbital, and blood samples were removed from the postcaval vein with a heparinized needle attached to a heparinized syringe. The blood samples were centrifuged at 1,200 × *g* for 10 minutes to obtain plasma, which were immediately frozen in liquid nitrogen and kept at –80°C until use.

Diazepam at 10 nmol was added to 1 mL of plasma as an internal standard. The sample was alkalinized with 0.1 mL of 5 M NaOH and extracted twice with 1 mL of n-hexane/methanol (4/1). The organic layer was extracted twice with 0.1 mL of 0.05% phosphoric acid. The extract was filtered through a 0.45-μm filter. A portion of the filtrate was subjected to high-performance liquid chromatography (HPLC) analysis.

Imipramine and desipramine were analyzed with HPLC-UV. An LC-10AD/SPD-10A system (Shimadzu, Kyoto, Japan) was used. Separation was performed on Gemini ODS (2.1 mm in inner diameter × 150 mm, Phenomenex, Torrance, CA, USA) with a precolumn (SecurityGuard, Phenomenex) at 40°C. The eluents were 80% 50 mM sodium phosphate buffer (pH 3.8), 20% acetonitrile. The flow rate was maintained at 0.2 mL/minute. Compounds were detected at 254 nm.

### Analysis of protein in the brain by western blotting

Rats were anesthetized with pentobarbital and sacrificed by decapitation. The brain was rapidly dissected out and divided into sections. The prefrontal cortex (PFC) and hippocampus were immediately frozen in liquid nitrogen and kept at –80°C until use.

The brain tissues were homogenized in 5 volumes of lysis buffer (50 mM Tris HCl buffer [pH 7.4], 150 mM NaCl, 1% Triton X-100, 0.5% sodium deoxycholate, 1% sodium dodecyl sulfate [SDS], 1 mM sodium orthovanadate, 1 mM NaF, 1 mM phenylmethylsulfonyl fluoride [PMSF], 2 μL/mL of protease inhibitor cocktail), and centrifuged at 20,000 × *g* for 20 minutes at 4°C. The protein contents of the supernatant were determined using the bicinchoninic acid (BCA) assay kit (Thermo Fisher Scientific, Waltham, MA, USA). An aliquot of the supernatant was used to prepare the SDS sample (1 μg protein/μL in SDS loading buffer). The SDS samples were incubated at 95°C for 2 minutes, and loaded at 10 μg protein onto 10% SDS-polyacrylamide gels. Proteins were separated at 60 V and then blotted to PVDF membranes in Tris-glycine transfer buffer at 100 V for 2 hours. The membrane was incubated with 5% skim milk in phosphate-buffered saline containing 0.1% Tween-20 (PBS-T) for 1 hour at room temperature, primary antibody overnight at 4°C, and horseradish peroxidase-conjugated secondary antibody for 1 hour at room temperature. The membrane was washed with PBS-T 3 times for 5 minutes after the incubation with the primary and secondary antibody. The membrane was developed with the ECL prime western blotting detection reagent (Amersham, Piscataway, NJ, USA), and analyzed using an imaging analyzer LAS-3000 (Fujifilm, Tokyo, Japan). Blots with anti-β-actin antibody were used as loading control.

### Statistical analysis

**Figure 1:**
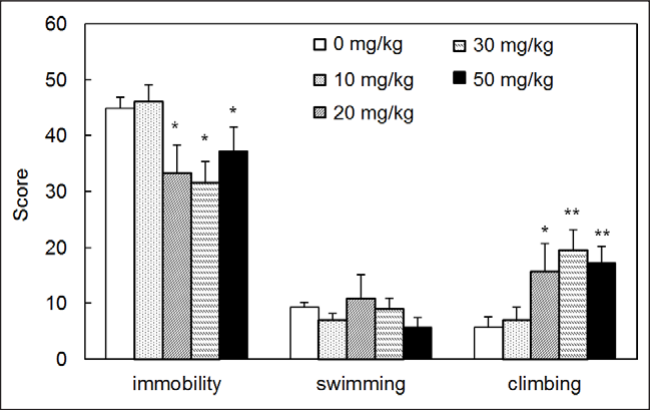
**Imipramine exerts dose-dependent antidepressant actions in rats.** The rats received imipramine intraperitoneally, after which they were subjected to the forced swim test. Each column represents the mean ± standard error of the mean (SEM) (n = 6 – 8). *p < 0.05, **p < 0.01 vs. control by Williams test.

**Figure 2:**
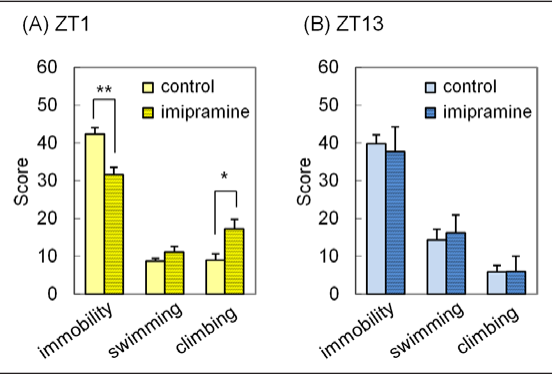
**Acute imipramine treatment produces dosing time-dependent effects in the forced swim test (FST).** The rats were administered imipramine at ZT1 **(A)** or ZT13 **(B)**. One hour after imipramine administration, the rats were subjected to the FST. Each column represents the mean ± standard error of the mean (SEM) (n = 10 – 13). *p < 0.05, **p < 0.01 by Student’s t-test.

**Figure 3:**
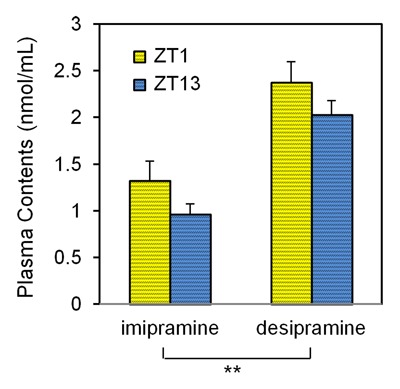
**Dosing time affects the plasma levels of imipramine and desipramine following acute imipramine treatment.** Imipramine (30 mg/kg) was administered at ZT1 or ZT13, and the plasma concentrations of imipramine and desipramine were measured 1 hour later. Each column represents the mean ± standard error of the mean (SEM) (n = 3). *p < 0.05 by 2-way analysis of variance (ANOVA).

**Figure 4:**
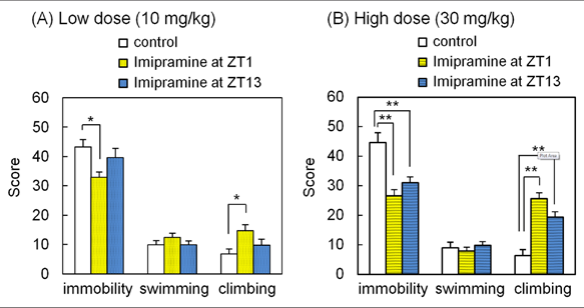
**Chronic imipramine treatment produces dosing time-dependent effects in the forced swim test (FST).** The rats were administered imipramine at ZT1 or ZT13 for 2 weeks, and then subjected to the FST. The effects of chronic imipramine treatment at 10 mg/kg **(A)** and 30 mg/kg **(B)** are shown. Each column represents the mean ± standard error of the mean (SEM) (n = 8 – 18). *p < 0.05, **p < 0.01 by Tukey’s test.

**Figure 5:**
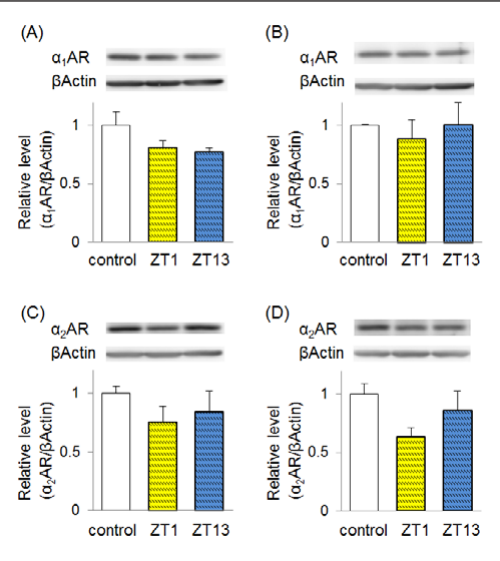
**Chronic imipramine treatment alters the protein level of adrenergic receptors.** Imipramine (30 mg/kg) was chronically administered at ZT1 or ZT13, and the protein levels of α_1_AR and α_2_AR were analyzed with western blotting. The levels of α_1_AR **(A, B)** and α_2_AR **(C, D)** in the prefrontal cortex (PFC) (A, C) and hippocampus (B, D) are shown. Each column shows the mean ± standard error of the mean (SEM) (n = 4). No statistically significant difference was detected between groups by Tukey’s test.

Data are expressed as the means ± standard error of the mean (SEM). Dose-dependent changes against the control group were analyzed using the Williams test (Figure 1). Differences between two groups were analyzed with Student’s t-test (Figure 2). Effects of two factors were analyzed using 2-way analysis of variance (ANOVA) (Figure 3). Multiple pairwise comparisons among three or more groups were analyzed with Tukey’s test (Figures 4 and 5). The significance level was set at p < 0.05. The JMP procedure (SAS Institute, Cary, NC, USA) was applied for statistical analysis.

## Results

### Effects of acute imipramine treatment on the FST

The effects of acute imipramine treatment at various doses on the FST are shown in Figure 1. In this experiment, imipramine was administered at ZT1. Williams test detected a significant reduction of immobility and a significant increase of climbing at doses equal to or higher than 20 mg/kg of imipramine (immobility: p = 0.0210, 0.0197, and 0.0275 for 20, 30, and 50 mg/kg, respectively; climbing: p = 0.0245, 0.0068, and 0.0078 for 20, 30, and 50 mg/kg, respectively). Since the effect at 30 mg/kg was larger than the effect at other doses, we used imipramine at 30 mg/kg in the following analysis.

The rats treated with imipramine at ZT1 or ZT13 were subjected to the FST in order to analyze the effect of dosing time on antidepressant activity. The results are shown in Figure 2. Imipramine significantly reduced immobility and increased climbing when administered at ZT1 (p = 0.0009 and p = 0.0126, respectively) (Figure 2A), but not at ZT13 (Figure 2B).

### Plasma concentration of imipramine

The plasma concentrations of imipramine and its metabolite, desipramine, following imipramine treatment are shown in Figure 3. Two-way ANOVA detected no interaction (F_1,8_ = 0.0013, p = 0.9719). The main effect in compound, imipramine and desipramine, was significant (F_1,8_ = 34.0154, p = 0.0004). Although the main effect in dosing time was not significant (F_1,8_ = 3.8042, p = 0.0869), both imipramine and desipramine were slightly increased at ZT1 compared to ZT13.

### Effects of chronic imipramine treatment on the FST

The effects of chronic imipramine treatment on the FST are shown in Figure 4. In this experiment, imipramine was administered at ZT1 or ZT13 for 2 weeks. We used two doses: the same dose as in the acute treatment (30 mg/kg), and the dose that was not effective in the acute treatment (10 mg/kg). At low dose, the ZT1 group showed 24% reduction in immobility and 116% increase in climbing against the control (p = 0.0126 and p = 0.0144, respectively) (Figure 4A). The differences between ZT1 and ZT13 were not significant (p = 0.1359 for immobility, p = 0.1778 for climbing). At high dose, significant differences in immobility and climbing were detected in the ZT1 and ZT13 groups against the control (p = 0.0001 and p = 0.0030 for immobility, p < 0.0001 and p = 0.0006 for climbing, respectively) (Figure 4B). The differences between ZT1 and ZT13 were not significant (p = 0.4310 for immobility, p = 0.0883 for climbing).

### Effects of chronic imipramine administration on adrenergic receptors

In order to investigate the alterations of the noradrenergic system induced by chronic treatment of imipramine, the protein levels of α_1_AR and α_2_AR were analyzed with western blotting (Figure 5). No significant differences were detected in the levels of α_1_AR and α_2_AR. However, the mean values of imipramine-treated groups were lower than those of the control. In particular, imipramine at ZT1 resulted in 37% reduction of α_2_AR in the hippocampus against the control, although the difference was not significant (p = 0.1018 vs. control, p = 0.6794 vs. ZT13).

## Discussion

The present study examined the chronopharmacological effects of acute and chronic imipramine treatment. We showed that imipramine works more efficiently in the morning than in the evening in rats. The antidepressant effect was observed in the morning (ZT1), but not in the evening (ZT13) with a single dose of 30 mg/kg of imipramine. The same trend was observed in the chronic treatment model. Chronic imipramine in the morning induced antidepressant effects in the FST even when the dose was lower than the effective dose in the acute treatment model. Chronic imipramine might increase the drug susceptibility of the rat brain most efficiently in the morning. To our knowledge, this is the first report that shows the chronopharmacological effects of an antidepressant in a chronic treatment model. Since antidepressants are usually used chronically in clinical settings, this model could be a useful model to analyze the chronopharmacological profile of antidepressants and the underlying mechanism of their chronopharmacological activity.

In the chronic treatment model, the rats were administered a low dose (10 mg/kg) of imipramine one hour before the FST as described in the Methods. Since imipramine was ineffective at this dose when administered acutely (Figure 1), the reduction of immobility and the increase of climbing observed in Figure 4 could not be because of the acute effect of the last low dose administration. This suggests that the chronic imipramine treatment caused these antidepressant effects. Chronic imipramine treatment might induce alterations of the adrenergic system (target system of imipramine) such as an enhancement of noradrenergic neurons’ susceptibility to imipramine, which would induce the antidepressant effects seen after the administration of a low dose of imipramine.

Chronopharmacological effects can be induced by the circadian fluctuation of the pharmacokinetics or pharmacodynamics of a drug [2]. Various drugs show dosing time-dependent pharmacokinetics [23,33,34,35]. Imipramine is metabolized to desipramine by CYP1A2, 3A4, and 2C19, and imipramine and desipramine are hydroxylated to the 2-OH form by CYP2D6 [36,37]. Both imipramine and desipramine have antidepressant activity, while the 2-OH form of both compounds does not have antidepressant activity. Therefore, intra-day fluctuation of the activity of CYP2D6 could explain the chronopharmacological action of imipramine. The expression of CYPs is controlled by biological clocks, and their expression and activity show a circadian rhythm [38,39,40,41,42,43]. The expression of CYP2D9, the murine homolog of human CYP2D6, shows a circadian rhythm with a peak at night in the mouse liver [43]. Figure 3 shows that the plasma concentrations of imipramine and desipramine were 17–37% higher at ZT1 than at ZT13. Although the difference (p = 0.0869) did not meet the criteria of statistical significance, we suspect that the circadian fluctuation of CYP2D9 might have caused the difference in imipramine concentrations between ZT1 and ZT13, which might contribute to the chronopharmacological action of imipramine to some extent.

Pharmacodynamics should also be considered. In brain monoaminergic systems, the contents of neurotransmitters and the expression of receptors and transporters show circadian rhythm [20,21,44]. Since imipramine exerts antidepressant activity by its interaction with the serotonergic and noradrenergic systems, the circadian rhythm of these neuronal systems could cause the chronopharmacological action of imipramine.

In the modified FST, the increase of swimming and climbing is related to the activation of serotonergic and noradrenergic activity in the brain, respectively [26]. Imipramine increased climbing in the present study, suggesting that the noradrenergic system should dominantly contribute to the antidepressant activity of imipramine in rats. We suspected that desipramine played a major role in this noradrenergic effect. Imipramine and desipramine inhibit both the serotonin transporter (SERT) and the noradrenaline transporter (NAT). However, the selectivity was different between imipramine and desipramine. The inhibitory action of imipramine is 27 times more potent on SERT than on NAT, whereas that of desipramine is 21 times more potent on NAT than on SERT [45]. Since the plasma concentration of desipramine was higher than that of imipramine (Figure 3), the effects of desipramine might appear dominantly in the FST.

Most antidepressants are effective only after chronic treatment in clinical settings. Chronic treatment with antidepressants induces alterations in the brain function, which may lead to the alleviation of depressive symptoms. The involvement of α_2_AR has been reported both in animal and clinical studies. Brain α_2_AR density is upregulated in patients with depression [30,46]. Chronic treatment with a tricyclic antidepressant induces the downregulation of α_2_AR in various brain regions of model animals [30,47]. The present results are consistent with those of previous studies. Although we could not detect significant differences, the protein levels of α_1_AR and α_2_AR tended to be reduced following chronic imipramine treatment (Figure 5). Comparing ZT1 and ZT3 groups, the reduction seemed larger in the ZT1 group, especially for α_2_AR in the hippocampus. Since α_2_AR exists in the presynaptic neurons and negatively modulates noradrenaline release [30], the downregulation of the α_2_AR might lead to increased noradrenergic neural transmission, which might induce antidepressant effects.

There are some concerns about the validity of the FST. In this study, a low dose (10 mg/kg) of imipramine was sufficient for an antidepressant effect in the chronic model; however, a higher dose was necessary to have an antidepressant effect in the acute model. These results are consistent with previous studies looking at other drugs. Amitriptyline, mianserin, desipramine, and fluoxetine have an antidepressant effect in the FST when chronically administered at a particular dose, but this same dose does not have an antidepressant effect when administered acutely [48,49]. Another study indicated that some drugs, such as mepyramine and promethazine, are effective with acute treatment but not with chronic treatment [50]. These findings suggest that the mechanism involved in the imipramine-induced anti-immobility effect in the FST is different in the chronic and acute models. Since chronic treatment is usually necessary for effective antidepressant therapy in clinical settings [24], the physiological changes observed in the acute model may be different from the changes seen in human depression. The FST used in the acute model may not be a good model for the analysis of the mechanisms involved in human depression. However, the acute FST is a useful tool for high throughput screening and analysis of antidepressant activity.

There are some inconsistencies about the chronopharmacological profile of antidepressant between the present results and those of previous studies. A tricyclic antidepressant, amitriptyline, shows circadian activity rhythm with a peak at early night in an acute mouse model [20]. A serotonin noradrenaline reuptake inhibitor (SNRI), milnacipran, induces a large increase in climbing at ZT13 rather than at ZT1 in an acute rat model [21]. In the present study, imipramine induced a large reduction in immobility and an increase in climbing in the morning (ZT1) in an acute rat model (Figure 2). We are unable to explain these inconsistencies based on the available data. However, we suspect that differences in the mechanism of action might contribute to the different chronopharmacological profiles. Although amitriptyline, milnacipran, and imipramine inhibit both SERT and NAT, the relative activity to SERT and NAT is different among these compounds [45]. Since serotonergic and noradrenergic systems show circadian activity rhythms with different peak times [21,44], the relative activity of different antidepressants on these neurons may be an important factor that determines their chronopharmacological profiles.

There are limitations to the present study. (1) The chronopharmacological effect was analyzed with only two time points. (2) The contributions of neuronal systems other than the noradrenergic system were not addressed. (3) Antidepressant activity was evaluated only with the FST and by using normal naïve rats. Firstly, it is impossible to determine the exact time point at which imipramine shows maximal antidepressant activity during the day. Although we observed higher antidepressant activity in the morning than in the evening, the data could not exclude the possibility that imipramine shows maximal antidepressant activity during the afternoon or at midnight. Chronopharmacological analysis with three or more time points should be important to discuss the precise chronopharmacological profiles of imipramine and other antidepressants, as well as to clarify the relationships between the chronopharmacological profile and the mechanism of action of antidepressants. Secondly, the present study analyzed the effect of chronic imipramine administration only on the adrenergic receptors. However, other neuronal systems such as the serotonergic, dopaminergic, and glutamatergic systems may also play an important role in the action of antidepressants [51]. We could not exclude the possibility that these systems show larger circadian fluctuations of the activity compared to the adrenergic system, and that these fluctuations are crucial in the chronopharmacological action of imipramine. The effects of antidepressants on these systems and their circadian system functionality should be considered. Thirdly, the chronopharmacological effects of antidepressants in depression patients or depressive-like animals are still unknown. Some physiological functions in depressive-like animals may be impaired and different from those in normal animals, which may alter the chronopharmacological effects of the antidepressants. Some drugs cannot induce antidepressant effects in animal models of depression even though they have antidepressant effects in normal animals [52,53]. Patients with depression and animal models of depression sometimes show disturbances in circadian system functionality [6,7,8,54,55,56]. Chronopharmacological profiles of antidepressant activity in depressive-like animals could be different from those in normal animals. Analyses using animal models of depression may clarify the chronopharmacological effects of antidepressants on behavior and brain monoaminergic systems in the depression.

## Conclusion

In this study, we revealed the chronopharmacological activity of imipramine in rats. In particular, we found that chronic imipramine treatment produced dosing time-dependent antidepressant effects at a dose that was ineffective when given acutely. In the FST, imipramine showed greater antidepressant effects in the morning than in the evening. Imipramine could interact with noradrenergic neurons more effectively in the morning than in the evening, and the chronic interaction with the neurons might induce the alterations of the receptor expression, which might explain the dosing time-dependent antidepressant effects of imipramine. This low-dose chronic model using the FST might be a useful model for the chronopharmacological analysis of antidepressants.
